# The effectiveness of online acceptance and commitment therapy-based interventions on depression, burnout, anxiety and stress in occupational contexts: A systematic narrative review

**DOI:** 10.1016/j.invent.2026.100909

**Published:** 2026-01-23

**Authors:** Veera Sofia Lampinen, Ella Kämper, Viktória Roxána Balla, Nina Katajavuori, Henna Asikainen

**Affiliations:** a Centre for University Teaching and Learning (HYPE), Faculty of Educational Sciences, University of Helsinki, Siltavuorenpenger 5A, FI-00014, Helsinki, Finland; b Cognitive Brain Research Unit, Department of Psychology and Logopedics, Faculty of Medicine, University of Helsinki, Haartmaninkatu 3, FI-00290, Helsinki, Finland

**Keywords:** Psychological flexibility, Workplace well-being, ACT, Online interventions, Burnout, Occupational mental health

## Abstract

Declining employee mental health has led to an interest in Acceptance and Commitment Therapy (ACT)-based interventions that aim to develop psychological flexibility. While existing literature primarily focuses on face-to-face interventions, evidence on online ACT interventions in occupational settings remains limited. This systematic narrative review synthesized findings on the outcomes of online ACT-based interventions for employees. Following the Preferred Reporting Items for Systematic Reviews and Meta-Analyses (PRISMA) 2020 and Synthesis Without Meta-analysis (SWiM) guidelines, six databases were searched, yielding 11 randomized controlled trials targeting burnout, depression, anxiety, and stress. Most studies reported modest but significant improvements in these outcomes, often sustained at follow-up. Psychological flexibility consistently emerged as the principal mechanism of change. Intervention formats and engagement varied widely; partially guided programs demonstrated higher adherence than fully self-guided formats, though time constraints and workload frequently hindered participation. Online ACT-based interventions appear tentatively promising for improving employee well-being, but methodological heterogeneity, limited cultural diversity, and short follow-up periods constrain firm conclusions. Future studies should employ standardized protocols, process-based measures, and longer follow-ups to clarify the mechanisms and sustainability of change.

## Highlights

• Online ACT interventions show tentative promise in reducing depression, anxiety, stress, and burnout.
• Psychological flexibility is identified as central to change in interventions.
• Intervention effects can strengthen during follow-up periods.
• Organizational support is crucial for intervention engagement.
• Variability in methodologies limits the strength of conclusions.

## 1 Introduction

Attention to the declining employee mental health is at an all-time high among researchers, employers, and popular media (Hammer et al., 2024; Lacerenza et al., 2024), exacerbated by the impact of the COVID-19 pandemic (Li et al., 2021; Sevov et al., 2025). Depression and anxiety are among the most prevalent mental health conditions, affecting 280 and 301 million people globally, respectively (World Health Organization, 2023a, World Health Organization, 2023b). In parallel, occupational burnout and stress remain widespread concerns, with stress functioning as both an outcome and a risk factor for burnout, depression, and anxiety (Maslach, 2003; Sun et al., 2012; World Health Organization, 2024; Koutsimani et al., 2019; Schonfeld and Bianchi, 2021). Collectively, depression, anxiety, burnout, and stress represent some of the most prevalent and costly threats to employee well-being and organizational functioning.

One promising solution to address this is based on Acceptance and Commitment therapy (ACT), a psychological intervention framework where the central aim is to develop individuals' psychological flexibility (Hayes et al., 2006). Psychological flexibility refers to the overarching capacity to remain open to inner experiences, stay present in the moment, and engage in behaviors that are consistent with personal values, even when faced with challenging situations, thoughts, and emotions (Kashdan and Rottenberg, 2010; Hayes et al., 2006). More specifically, psychological flexibility consists of six interrelated core processes (Hayes et al., 2011). First, acceptance indicates willingness to experience uncomfortable or unwanted thoughts, feelings, and sensations without avoiding or suppressing them. The second, cognitive defusion, is related to learning to view thoughts as transient mental events rather than literal truths or commands that always dictate behavior. Third, present moment awareness signifies learning to direct attention to the here and now instead of the past or future. Fourth, self-as-context indicates a conception of the self as an observer of experiences, thoughts, and feelings, distinct from the content of those. Fifth, values pertain to recognizing and connecting with what matters the most to an individual; that is, finding what is meaningful and purposeful. Finally, committed action reflects being able to choose and make choices guided by values, even in the face of challenges.

Online workplace interventions have most often drawn on cognitive behavioral therapy (CBT; Phillips et al., 2019) and mindfulness-based stress reduction (Taylor et al., 2022), both of which have shown promise in supporting employee well-being (Stratton et al., 2022; Cameron et al., 2025). Compared to CBT, which focuses on restructuring maladaptive thoughts, ACT emphasizes acceptance and flexible engagement with internal experiences, with the overall aim of redirecting attention from avoidance to values-congruent behavior, thereby shifting emphasis from symptom reduction to a function-oriented approach (Anusuya and Gayatridevi, 2025; Finnes et al., 2019; Hayes et al., 2013). While conceptually related to mindfulness approaches, ACT also embeds mindfulness in a broader framework that explicitly links present-moment awareness to values-based action and psychological flexibility (Davis et al., 2015; Hayes et al., 2006).

Meta-analyses have previously reviewed online ACT without an explicit focus on employee populations (e.g., Han and Kim, 2022), reporting modest improvements in depressive symptoms, anxiety, and stress. However, employees may often access digital ACT programs as lower-threshold, preventive resources rather than clinical treatment, and must engage with them alongside competing work demands and time pressure (Carolan and de Visser, 2018; Paterson et al., 2024; Yarker et al., 2022). These contextual factors differentiate employee populations from symptom-targeted samples in prior reviews, warranting a synthesis that integrates findings across occupational studies.

Previous research has conducted review studies about the effects of ACT interventions targeting different mental health issues in a plethora of occupational contexts (e.g., Prudenzi et al., 2022; see Unruh et al., 2022 for meta-analysis; Towey-Swift et al., 2023; Vega-Campos et al., 2025 for reviews), where ACT-based interventions have also demonstrated potential to effectively promote well-being (Unruh et al., 2022; Russo et al., 2025). However, in such studies, the interventions have been carried out primarily face-to-face (Oakman et al., 2018; Vega-Campos et al., 2025), and we still do not fully understand the potential of online ACT interventions on occupational well-being.

Although a growing number of studies specifically explore online ACT in employee samples (e.g., Barrett and Stewart, 2021; Zhang et al., 2024), their findings remain scattered across diverse delivery formats and outcome measures, making it difficult to derive an overall understanding from individual trials alone. A focused synthesis is therefore needed to summarize how online ACT has been studied in workplace settings and clarify what is currently known about its potential in addressing burnout, depression, anxiety, and stress. This may also yield practical insights into more effective ways of supporting occupational well-being.

Thus, the present review focuses specifically on online workplace ACT interventions, with particular attention to their delivery to explore their effectiveness and potential for application. We adopt a systematic narrative review design guided by Synthesis Without Meta-analysis reporting standards (SWiM) (Campbell et al., 2020). This approach allows for the transparent and structured synthesis of findings when pooling effect sizes is complex, while also accommodating variation in study design, delivery format, and workplace context. By doing so, the review can provide a comprehensive overview of how online ACT interventions have been tested in occupational settings and the extent to which they may impact depression, anxiety, stress, and burnout. This review had four main objectives: (1) to synthesize evidence on the effects of online ACT interventions in workplace settings on depression, anxiety, burnout, and stress, and assess studies' methodological quality; (2) to examine how these interventions have been delivered and studied, including format, engagement and level of guidance; (3) examine how psychological flexibility is conceptualized in the interventions in relation to intervention-related change, and (4) to identify strengths, limitations and research gaps in the current literature.

## 2 Method

Given the heterogeneity of study and intervention designs (See Table 4), as well as outcomes, a quantitative meta-analysis was not feasible. Instead, we conducted a systematic narrative review, using SWiM (Campbell et al., 2020) and the Preferred Reporting Items for Systematic reviews and Meta-Analyses (PRISMA) 2020 guidelines (Page et al., 2021).

### 2.1 Search strategy and selection criteria

**Fig. 1:**
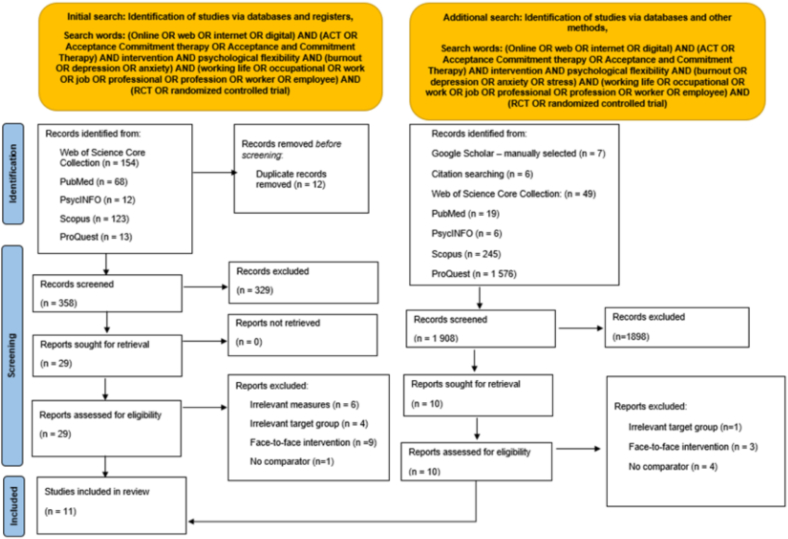
PRISMA Flowchart depicting search strategy and screening.

The search strategy and screening were conducted according to PRISMA 2020 guidelines (Page et al., 2021) (See Fig. 1). Population, Intervention, Comparator, Outcome (PICO) (Eriksen and Frandsen, 2018) were used to inform the eligibility criteria (See Table 1). For full search strings for both searches, see the PRISMA flowchart (Fig. 1) and Appendix B.

**Table 1:** The inclusion and exclusion criteria grouped into Population, Intervention, Comparison, and Outcome (PICO) categories.

	Include	Exclude
**Population**	Initial & additional search: Described as a staff group in any profession, full-time or part-time.	Employment status is not described or considered.
**Intervention**	Initial & additional search: Based on the principles of ACT, ACT is mentioned as a basis for the intervention. >75% digital delivery (e.g., web platforms, apps, digital modules, Teams/Zoom sessions).	ACT is not explicitly mentioned as the basis for the intervention. >25% face-to-face delivery that acts as the main component of the intervention (e.g., the intervention is primarily based on face-to-face sessions).
**Comparison**	Initial & additional search: There is a comparator of any format, and participants have at least been attempted to be randomly assigned into intervention and control groups.	There is no comparator and/or no attempt to randomly assign participants to intervention and control groups.
**Outcome**	Initial search: Either burnout, depression, and/or anxiety were assessed by any validated tool as an outcome measure. Additional search: Either burnout, depression, anxiety, and/or stress were assessed by any validated tool as an outcome measure.	Burnout, depression, and/or anxiety have not been measured, or no validated tool has been used. Burnout, depression, anxiety, and/or stress have not been measured, or no validated tool has been used.

Note. ACT= Acceptance and commitment therapy.

Six databases were employed for the initial search of articles in May 2024: PubMed, Web of Science, PsycINFO, Medline, Scopus, and ProQuest. The objective for the review was to examine the effectiveness of online ACT-based interventions. Our theoretical framework was built on psychological flexibility, a central construct in ACT, as a mitigating factor for (occupational) well-being.

Our initial search strategy focused on psychological outcomes that are prevalent in occupational contexts according to the literature, namely depression, anxiety, and burnout, as primary outcome measures. However, after conducting the preliminary search, we identified a limited number of studies meeting our inclusion criteria (*N* = 9). Given the relationship between stress, burnout, depression, and anxiety, where stress is recognized as both a precursor and a contributing factor to these conditions, especially for burnout, we refined our inclusion criteria to incorporate stress as an additional outcome measure (Maslach and Leiter, 2016; Melamed et al., 2006).

Thus, we conducted an additional search incorporating the revised criteria in March 2025. This search was performed using the same databases as in the original search. Additionally, Google Scholar was used due to its suitability as a complementary tool in systematic reviews because it includes grey literature that may not be available in traditional academic databases (Haddaway et al., 2015; Bramer et al., 2017). Furthermore, citation searching from the already included articles, as well as re-reviewing the excluded articles from the original searches in light of the revised criteria was conducted to identify any additional eligible studies.

Studies were included from both searches if they met the eligibility criteria set by the authors based on the PICO framework. In brief, eligible studies examined employed staff in any profession, evaluated ACT-based interventions primarily delivered online, included a comparator condition with at least attempted randomization, and reported outcomes related to burnout, depression, anxiety, or stress using validated measures. Only studies reported in English were considered. Full details of inclusion and exclusion criteria are presented in Table 1.

### 2.2 Screening and study selection

In the initial search, studies were screened blind by three independent reviewers on Rayaan (https://www.rayyan.ai/), and disagreements were resolved with discussion between the reviewers (disagreement rate: 4%). The inclusion process included two stages. In the screening stage (1), all records were screened for eligibility based on their abstract and title, and all duplicates were removed. The eligibility stage (2) included acquiring full-text versions of the articles and checking their eligibility against the criteria (see Table 1) again. In the additional search, studies were screened by one author; however, final decisions on the additional inclusions were discussed and agreed upon by all authors. During screening, the titles and abstracts identified through the search were reviewed. Articles that met the selection criteria were screened and then selected for full-text review.

### 2.3 Data extraction

Data from the articles were extracted based on key methodological variables for tabulation: Authors (year); participants; randomization; attrition rates at post-intervention; measurement points; comparators; outcomes; results from relevant outcomes (See Table 3); demographic reporting (See Table 2), and intervention format and contents (See Table 4). The data was extracted by the first author and was subsequently reviewed by all authors.

**Table 2:** Summary of participant demographics.

Authors (year)	Age (*M*)	Gender (female)	Gender (male)	Ethnicity
Barrett and Stewart (2021)	37.1	37	5	Not reported
Fiery (2016)	40.4	108	10	White: 103 Black: 1 Asian: 2 Other: 10
Hofer et al. (2018)	3.8	83	36	Not reported
Lappalainen et al. (2013)	43.4	0	23	Not reported
Lu et al. (2023)	35.3	141	4	Not reported
Otared et al. (2021)	33.4	19	21	Not reported
Puolakanaho et al. (2020)	46.9	133	35	Not reported
Taylor et al. (2023)	36.9	222	183	White: 357 Asian: 22 Black: 13 Mixed: 13
Vahabi et al. (2022)	38.4	Not reported	Not reported	Not reported
Zhang et al. (2024)	38.5	88	20	Not reported
Sasaki et al. (2023)	36.6	841	0	Not reported
Total reported (%):	**39.2**	**1672 (83.2%)**	**337 (16.8%)**	**521** **White: 460 (88.3%)** **Asian: 24 (4.6%)** **Black: 14 (2.7%)** **Mixed: 13 (2.5%)** **Other: 10 (1.9%)**

*Note. M* = mean.

For the Cochrane (V2) Risk of Bias assessment tool (Sterne et al., 2019), data from five domains were additionally extracted for risk of bias evaluations: (1) potential bias arising from the randomization process (e.g., allocation concealment); (2) potential bias due to deviations from intended interventions (e.g., adherence monitoring); (3) potential bias due to missing outcome data (e.g., handling of missing data); (4) potential bias in measurement of the outcome (e.g., blinding of outcome assessors); and (5) potential bias in selection of the reported result (e.g., selective reporting). The data for the risk of bias assessment were independently extracted by three authors.

### 2.4 Synthesis approach

Due to variation in study designs, outcome measurement, and reporting formats, it was not feasible to pool effect sizes in a meta-analysis. Instead, we applied a systematic narrative synthesis guided by the SWiM reporting guidelines (Campbell et al., 2020).

Studies were grouped according to delivery format (self-guided and partially guided), outcome domains (burnout, depression, anxiety, stress), and population type (occupational sector, demographics) to facilitate comparison across heterogeneous designs. Data were presented in structured summary tables (study characteristics, intervention format, outcomes, attrition, demographics; See Table 2, Table 3, Table 4).

**Table 3:** Summary and key characteristics of each study.

Authors (year)	Participants	Randomization	Attrition at post-intervention (from randomized sample)	Measurement points	Comparator(s)	Outcomes	Results from relevant outcomes
Barrett and Stewart (2021)	*N* *=* 42 Intervention = 22 Control = 20 Social and healthcare professionals Ireland The UK The USA Philippines	Random allocation using an online random number generator.	*N* *=* 16 ACT intervention = 9 Comparator (CBT intervention) = 7,	Pre-intervention, post-intervention	CBT-intervention	**PSS** GHQ-12 **MBI** WAAQ	Burnout decreased significantly from baseline to post-intervention in both interventions. Perceived stress decreased significantly from baseline to post-intervention in both interventions. No significant between-group differences in burnout or perceived stress scores between ACT and CBT interventions.
Fiery (2016)	*N* *=* 119 Intervention = 60 Control = 59 Animal shelter staff USA	Random allocation from those who completed 75% of the baseline survey, Random sample function in SPSS.	*N* *=* 66 Intervention = 42 Control = 22	Pre-intervention, weekly, post-intervention, 6-week follow-up	Control (waitlist)	SCS-SF DISC 2.1 **STSS** UWES AAQ-II WAAQ **MBI (emotional exhaustion subscale)** Measured at baseline only: I-PANAS-SF FFMQ-SF	No significant effect of time on emotional exhaustion subscale (MBI) at post-intervention nor 6-week follow-up. No significant group x time interaction for emotional exhaustion (MBI) at post-intervention nor at 6-week follow-up. No significant effect of time on secondary traumatic stress at post-intervention nor at 6-week follow-up. No significant group x time interaction for secondary traumatic stress at post-intervention nor at 6-week follow-up.
Hofer et al. (2018)	*N* *=* 119 Intervention = 61 Control = 58 Various occupational fields Switzerland and Germany	Block randomization (2:1:1 ratio), computer-generated random sequences.	*N* = 10 Intervention = 8 Control = 2	Pre-intervention, post-intervention, 3-month follow-up	Control (waitlist)	**PSS** MHC-SF DERS **MBI** **BDI-II** **AAQ-II** CFQ KIMS	Burnout decreased significantly from baseline to post-intervention and compared to the control group. Effects sustained and strengthened at 3-month follow-up. Perceived stress decreased significantly from baseline to post-intervention compared to the control. Effects sustained and strengthened at 3-month follow-up. Depression decreased significantly from baseline to post-intervention and compared to the control group. Effects sustained and strengthened the 3-month follow-up.
Lappalainen et al. (2013)	*N* = 24 Intervention = 11 Control = 12 Various occupational fields (not specified) Finland	Divided into pairs based on BDI scores, the order of pairs was randomized, and randomly allocated to intervention or control.	*N* *=* 1 Intervention = 1	Pre-intervention, post-intervention, 6-month follow-up	Control (waitlist)	GSI VAS ERI **BDI** **BBI-15** AAQ-II	Marginally significant decrease in depression scores in the intervention group compared to the control group, group x time interaction (*p* = .072). Depression scores decreased significantly within-group from post-intervention at 6-month follow-up. No significant decrease in the burnout group compared to the control (no significant group x time interaction), burnout decreased significantly within-group from baseline to post-intervention. Burnout scores decreased significantly within-group at 6-month follow-up.
Lu et al. (2023)	*N* = 145 Intervention = 72 Control = 73 Nurses China	Randomization using computer-generated sequences by independent researchers, concealed allocatiand on, and data collectors were blinded to group assignments.	*N* = 12 Intervention = 4 Control = 8	Pre-intervention, primary measures weekly, post-intervention, 3-month follow-up	Control (waitlist)	Pre- and post-intervention: PSQI WPS **MBI-HSS** **GAD-7** **PHQ-9** AAQ-II CFQ MAAS VQ Weekly: **PHQ-9** **GAD-7** Follow-up: **PHQ-9** **GAD-7** **MBI-HSS** WPS PSQI	Significant decrease in depression scores in the intervention group compared to the control group, significant group × time interaction at post-intervention. Between-group effects sustained and strengthened at 3-month follow-up. Significant decrease in burnout scores in the intervention group from baseline to post-intervention, with a significant group × time interaction. Between-group effects sustained and strengthened at 3-month follow-up. Significant decrease in anxiety scores in the intervention group compared to the control, significant group × time interaction at post-intervention. Between-group effects sustained at 3-month follow-up.
Otared et al. (2021)	*N* = 40 Intervention = 20 Control = 20 Healthcare workers Iran	Randomly allocated to treatment/ control. Randomization method not specified.	None reported	Pre-intervention, post-intervention	Control (waitlist)	QOLI GAF **BDI-II** **BAI** AAQ-II	Significant decrease in depression scores in the intervention group compared to the control group at post-intervention Significant decrease in anxiety scores in the intervention group compared to the control group at post-intervention.
Puolakanaho et al. (2020)	*N* = 168 Intervention = 88 Control = 80 Various occupational sectors Finland	Matched-pair randomization based on sex, age, and education, followed by simple randomization within pairs.	*N* = 33 Intervention = 15 Control = 18	Pre-intervention, post-intervention, 6-month follow-up, 12-month follow-up	Control (treatment-as usual)	**PSS-10** WAQ SCL-90 **BBI-15** **DASS-D DASS-A** **AAQ-II** **DASS-S** LSQ RYFF KEYES FFMQ ATQ-B ATQ-F PSYFLE	Significantly different decrease in burnout scores in the intervention group compared to the control group at post-intervention. Between-group effects sustained at 6 months follow-up, sustained and strengthened at 12-month follow-up. Significantly different decrease in stress scores in the intervention group compared to the control group at post-intervention. Between-group effects were sustained at 6- and 12-month follow-up. Significantly different decrease in depression scores in the intervention group compared to the control group at post-intervention. Between-group effects were sustained at 6- and 12-month follow-up. Significantly different decrease in anxiety scores in the intervention group compared to the control group at post-intervention. Between-group effects were sustained at 6- and 12-month follow-up.
(Taylor et al. (2023)	*N* = 405 ACT intervention = 100 CBT intervention = 101 BA intervention = 102 Control = 102 Various occupational sectors UK	Randomly allocated to different interventions and a control group via Qualtrics software	*N* = 21 ACT intervention: 11 CBT intervention: 6 BA intervention: 4	Pre-intervention, post-intervention, 1-month follow-up	Control (waitlist), CBT intervention, BA intervention	**PHQ-8** **GAD-7** SWEMWBS Unmind Index WPAI MARS	Significant decrease in depression scores in the ACT intervention group compared to the control group, significant group × time interaction at post-intervention. Between-group effects sustained but attenuated at 1-month follow-up. Significant decrease in anxiety scores in the intervention group compared to the control group, significant group × time interaction at post-intervention. Between-group effects sustained but attenuated at 1-month follow-up. The ACT intervention showed similar reductions in depression scores compared to BA and CBT interventions, with no significant differences between the active interventions at post-intervention.
Vahabi et al. (2022)	*N* = 29 Intervention = 17 Control = 12 Migrant live-in caregivers Canda	Randomized into intervention and control using a random number generator.	*N* = 7 Intervention: 1 Control: 6	Pre-intervention, post-intervention, 6-week follow-up	Control group (waitlist)	AAQ-II **DASS-A** **DASS-D** **DASS-S** CAMS-R MSMR-I	No significant decrease in depression scores in the intervention group compared to the control, no significant group × time interaction at post-intervention. No significant effects at 6-week follow-up. No significant decrease in anxiety scores in the intervention group compared to the control, no significant group × time interaction at post-intervention. No significant effects at 6-week follow-up. No significant decrease in stress scores in the intervention group compared to the control group from pre- to post-intervention. Significant within-group decrease in stress scores at 6-week follow-up.
Zhang et al. (2024)	*N* = 108 Intervention = 54 Control = 54 Healthcare professionals China	Simple 1:1 randomization by blinded researchers, who generated sequences on a website where participants were assigned to a correct group. Data collectors were unaware of group assignments.	*N* = 40 Intervention: 19 Control: 21	Pre-intervention, post-intervention, 1-month follow-up	Control (waitlist)	**DASS-21** **MBI** compACT	Significant decrease in depression scores in the ACT intervention group compared to the control group, significant group × time interaction at post-intervention. Sustained at 1-month follow-up. Significant decrease in anxiety scores in the ACT intervention group compared to the control, significant group × time interaction at post-intervention. Sustained at 1-month follow-up. Significant decrease in stress scores in the ACT intervention group compared to the control, significant group × time interaction at post-intervention. Sustained at 1-month follow-up. Significant decrease in burnout scores in the ACT intervention group compared to the control, significant group × time interaction at post-intervention. Sustained at 1-month follow-up.
Sasaki et al. (2023)	841 Intervention = 424 Control = 417 Various occupational sectors Japan	Stratified permuted-block random table by an independent biostatistician. The random allocation sequence was computer-generated, with a fixed block size of 4. Researchers blinded.	*N* = 468 Intervention: 279 Control: 189	Pre-intervention, post-intervention, 3-month follow-up, 6-month follow-up	Control (waitlist)	PWBS **K6** ES	In the intention-to-treat analysis (all randomized participants), no significant decrease in psychological distress was observed in the ACT intervention group compared to the control. No significant effects at post-intervention or at 3- and 6-month follow-up. In the per-protocol analysis (participants who completed the intervention), psychological distress scores decreased significantly in the ACT group compared to the control group at post-intervention. Between-group effect maintained at three-month follow-up; no longer significant at six months.

Note. PSS= Perceived Stress Scale; GHQ-12 = General Health Questionnaire-12; MBI = Maslach Burnout Inventory; WAAQ = Work-related Acceptance and Action Questionnaire; SCS-SF = Self-Compassion Scale – Short Form; DISC2.1 = Demand-induced strain compensation 2.1; STSS = Secondary Traumatic Stress Scale; UWES = Utrecht Work Engagement Scale; AAQ-II = Acceptance and Action Questionnaire II; I-PANAS-SF = International Positive and Negative Affect Schedule – Short Form; FFMQ-SF = Five Facet Mindfulness Questionnaire – Short Form; MHC-SF = Mental Health Continuum – Short Form; DERS = Difficulties in Emotion Regulation Scale; BDI-II = Beck Depression Inventory – Second Edition; CFQ = Cognitive Fusion Questionnaire; KIMS = Kentucky Inventory of Mindfulness Skills; GSI = Global Severity Index; VAS = Visual Analogue Scale; ERI = Effort-Reward Imbalance Questionnaire; BBI 15 = Bergen Burnout Indicator – 15 item; PSQI = Pittsburgh Sleep Quality Index; WPS = Work Productivity Survey; MBI-HSS = Maslach Burnout Inventory – Human Services Survey; GAD-7 = Generalized Anxiety Disorder 7-item scale; PHQ-9 = Patient Health Questionnaire – 9 item; MAAS = Mindful Attention Awareness Scale; VQ = Valuing Questionnaire; QOLI = Quality of Life Inventory; GAF = Global Assessment of Functioning; BAI = Beck Anxiety Inventory; PSS-10 = Perceived Stress Scale – 10 item; SCL-90 = Symptom Checklist-90; DASS-D = Depression Anxiety Stress Scales – Depression subscale; DASS-A = Depression Anxiety Stress Scales – Anxiety subscale; DASS-S = Depression Anxiety Stress Scales – Stress subscale; LSQ = Life Satisfaction Questionnaire; RYFF = Ryff Scales of Psychological Well-Being; KEYES = Scales of Social Well-being; FFMQ = Five Facet Mindfulness Questionnaire; ATQ-B = The Automatic Thoughts Questionnaire – Believability; ATQ-F = The Automatic Thoughts Questionnaire – Frequency; PSYFLE = Skills Related to Psychological Flexibility; SWEMWBS = Short Warwick-Edinburgh Mental Well-being Scale; Unmind Index = Unmind Mental Health Index; WPAI = Work Productivity and Activity Impairment Questionnaire; MARS = Medication Adherence Rating Scale; CAMS-R = Cognitive and Affective Mindfulness Scale – Revised; MSMR-I = Mindful Self-Management Rating Inventory; DASS-21 = Depression Anxiety Stress Scales – 21 item; compACT = Comprehensive assessment of Acceptance and Commitment Therapy processes; PWBS = Ryff's Psychological Well-Being Scale; K6 = Kessler Psychological Distress Scale – 6 item; ES = Euthymia Scale. Bolded abbreviations in the Outcomes column represent outcomes relevant to this review.

**Table 4:** Summary of each study's intervention structure and contents.

Authors *(Intervention name)*	Intervention delivery platform	Support level	Duration	Target population	Intervention structure and core content	Intervention session themes
Barrett and Stewart (2021) *Stress Management Programme*	Completely online, web-based	Self-guided	2 weeks	Social and healthcare workers internationally	3 pre-recorded video ACT-based sessions, including content, exercises, and between-session homework. Emphasis on using the 6 ACT core processes for stress management.	1. Control and Awareness (experiential avoidance, cognitive fusion) 2. Acceptance and Values (cognitive fusion, self-as-context, values, acceptance) 3. Acceptance and Action (values, committed action)
Fiery (2016)	Completely online, audio recordings delivered via email	Self-guided	4 weeks	Animal shelter workers in the USA	3 weekly 20-min ACT-consistent pre-recorded audio meditations. Daily practice encouraged. In week 4, participants selected one of the three meditations for daily listening practice. Emphasis on self-compassion and ACT core processes on burnout and job engagement.	1. Compassionate body scan 2. Breath work, compassion, self, and others 3. Self-compassion for a personal experience of suffering
Hofer et al. (2018)	Completely online, web-based ACT self-help book with supplementary audio materials and worksheets	Self-guided	6 weeks	Various occupational sectors in Switzerland and Germany	11-chapter ACT self-help book delivered in 6 parts over 6 weeks with audio-guided exercises and worksheets. Emphasis on using the 6 ACT core processes for reducing stress and burnout.	1. Psychoeducation regarding burnout, stress, and ACT 2. Identifying undesirable experiences, cognitive defusion 3. Mindfulness, acceptance, and experiential avoidance 4. Self-as-context, self-concepts, committed action 5. Committed action, value-aligned goal setting, self-compassion
Lappalainen et al. (2013) *P4Well*	Hybrid, in-person group meetings and one feedback session, web-based portal, mobile application, and wearable devices	Partially guided	12 weeks	Various occupational sectors (not specified) in Finland	3 ACT-trained therapist-led group meetings spaced 4 weeks apart, 1 feedback session based on HRV measurements with an exercise physiologist, combined with continuous independent web- and mobile-based self-monitoring, exercises between meetings. Emphasis on using ACT to reduce stress and mood-related symptoms.	1. Introductory meeting; background, measurements, values, goals, mindfulness 2. Reflection on the current individual situation 3. Acceptance
Lu et al. (2023)	Completely online, mobile app (Rain Classroom/WeChat platform)	Self-guided	5 weeks	Nurses in China	5 weekly video-based ACT-based modules (15–30 min each) with worksheets and audio-guided exercises (10–20 min). Each module also included a thematic course content and homework. Emphasis on reducing anxiety and depression symptoms.	1. Opening ACT 2. Observing one's mind 3. Mindful living 4. Knowing what matters 5. Doing important things
Otared et al. (2021)	Completely online, live group videoconferencing platform	Partially guided	8 weeks	Healthcare workers in Iran	8 weekly 75-min ACT-trained therapist-led ACT group sessions. Emphasis on reducing anxiety and depression symptoms.	1: ACT foundations 2: Depression, anxiety, and ACT, strategies for inner experiences 3: Acceptance and experiential avoidance 4: Present-moment awareness 5: Cognitive fusion and defusion 6: Values clarification 7: Acceptance, mindfulness, committed action, self-as-context 8: Review and consolidation of ACT techniques and strategies
Puolakanaho et al. (2020)	Hybrid, face-to-face small group sessions and a web-based platform for home practice	Partially guided	8 weeks	Various occupational sectors in Finland	8 weekly small group sessions and daily home exercises. Emphasis on reducing burnout, stress, and depressive symptoms.	1. Differentiating oneself from one's thoughts and emotions and evaluating one's resources and the use of one's time. 2. Practicing observing without evaluation, defining one's values, and forming individual intervention objectives. 3. Experiencing the connection between mind and body and familiarizing oneself with reactions that emerge in difficult situations. 4. Recognizing the automaticity of thinking, distancing oneself from one's mind (own thoughts), and letting go of control efforts. 5. Learning to face difficulties with openness, empathy, and curiosity. 6. Power of practicing compassion and acceptance, clarifying one's own life and work values, and increasing value-based actions. 7. Investigating the connection between mood and daily routines and recognizing the sources of joy and gratitude. 8. Recognizing workable strategies for future use and defining reminders of being present in different contexts.
Taylor et al. (2023) *Finding Happiness*	Completely online, mobile app,	Self-guided	3 weeks	Various occupational sectors in the UK	7 online sessions, 10–18 min each, with exercises that are encouraged to be practiced outside of sessions. Emphasis on reducing depressive symptoms.	1. Examining behavior 2, Clarifying values 3. Designing experiments 4. Expanding sense of meaning and purpose⁎
Vahabi et al. (2022) *WE2CARE*	Completely online, web-based, 90-min live videoconferences	Partially guided	6 weeks	Migrant live-in caregivers in Canada	6 weekly learning modules introducing participants to the 6 core ACT processes, and weekly peer discussions facilitated by two researchers.	1. Reflecting on the present journey 2. Developing adaptive ACT strategies to deal with distressing experiences 3. Experiencing the transcendent self 4. Getting in touch with values and meaning 5. Building a supportive network 6. Committing to Valued Action
Zhang et al. (2024) *iACT*	Completely online, mobile app (WeChat)	Partially guided	6 weeks	Health care professionals in China	21 self-guided sessions pre-recorded by a psychologist and a meditation instructor, with a new session every other day. Each session (18–23 min) teaching video of a therapist, and exercises. Additionally, there were 7 web-based live sessions every 6 days by a therapist to review lessons, discuss questions, and potential challenges. Using ACT to reduce depressive symptoms.	1. Understanding human suffering 2. Search for the course of pain 3. Escaping pain causes problems 4. Brain mechanism of avoidance 5. Acceptance 6. Cultivate a positive attitude 7. Get out of your mind 8. Turn off the pain switch – cognitive fusion 9. Introduce cognitive defusion 10. The 3 senses of self 11. Being the observing self 12. Introduce mindfulness 13. Experience mindfulness practice 14. Sitting meditation 15. Practice mindful eating 16. Values as chosen life directions 17. What values are and are not 18. Choose your values 19. Make an action plan 20. Build patterns of effective action 21. Applying values to daily life
Sasaki et al. (2023) *Happiness Mom*	Completely online, web-based	Self-guided	12 weeks	Working mothers in various occupational sectors in Japan	8 learning modules, 15–30 min each. Using ACT to improve psychological well-being.	1. Well-being education 2. Acceptance and willingness 3. Defusion 4. Mindfulness and self-compassion 5. Self-as-context 6. Values 7. Committed actions 8. Wrap up

Note. ACT= Acceptance and Commitment Therapy. HRV = Heart rate variability. The 6 core ACT processes indicate: acceptance, cognitive defusion, present-moment awareness (or mindfulness), self-as-context, values, and committed action.

⁎ = Describes the general themes of the intervention, as themes for different sessions were not explicitly described or listed.

As outcome measures and reporting formats also varied substantially across studies, no common standardized metric was applied. Instead, we summarized the results narratively, retaining the original effect estimates, significance levels, and outcome scales as reported by the primary studies. This approach follows SWiM guidance for transparent reporting when synthesis relies on reported metrics rather than pooled effect sizes (Campbell et al., 2020).

Heterogeneity was examined narratively by comparing outcomes according to delivery type, attrition, and contextual factors. We followed a systematic, multi-step approach aligned with guidance on narrative synthesis in systematic reviews (Popay et al., 2006; Popay et al., 2017). First, each included study was reviewed in detail, with key study characteristics, contextual information, and outcome trends extracted (see Table 2, Table 3, Table 4). In the second step, data were inductively coded into conceptual categories that related to the research question, including intervention delivery format, occupational context, participant engagement, and reported outcomes.

Themes were color-coded so that blue represented static study context (e.g., inclusion criteria), green indicated outcomes (e.g., effect sizes for burnout), yellow signified theoretical mechanisms (e.g., psychological flexibility), and pink indicated methodological details (e.g., questionnaires used). Color-coding was carried out manually on the full-text articles on Avidnote (https://app.avidnote.com/), where articles were stored, and also went beyond tabulated data by incorporating qualitative insights drawn directly from the results and discussion sections of each study to support thematic depth and contextual interpretation. In the third step, similar codes were manually grouped into higher-order descriptive themes with pen and paper that reflected recurring patterns or contrasts across the data set. In the final step, themes were written out, reviewed, refined, and discussed among the author team.

To explore attrition rates and potential patterns based on intervention format, we calculated the percentage of participants who dropped out of each intervention group at post-intervention and subsequently looked at the attrition percentages for self-guided and partially guided interventions (See Table 3). Weighted average attrition rates were then calculated separately for self-guided and partially guided interventions by multiplying each study's intervention group dropout percentage by the number of participants assigned to that group, summing these values, and dividing by the total number of intervention participants across all relevant studies.

Methodological quality was assessed with the Cochrane (V2) Risk of Bias (RoB 2) assessment tool (Sterne et al., 2019). The RoB 2 employs a structured approach with predefined signaling questions along with an algorithm to help guide assessors with bias judgments. The tool assesses five key domains where bias may arise: (1) bias arising from the randomization process, (2) bias due to deviations from intended interventions, (3) bias due to missing outcome data, (4) bias in measurement of the outcome, and (5) bias in selection of the reported result. Domain questions have answer options “Yes”, “Probably Yes”, “No Information”, “No”, and “Probably No”. Based on the answers to these questions, each domain is rated as “Low risk”, “Some concerns”, and “High risk”. Based on the assessments in each domain, studies are additionally given an overall risk of bias rating. Assessors document all answers and rationale for judgments.

We employed an independent reviewing process, where three authors assessed all studies with the RoB 2 tool. All reviewers independently assessed each study's relevant outcomes. After each author completed assessments, we compared judgments, and disagreement was solved by discussion between the three authors where necessary (for a breakdown of the original judgments from each author, see Appendix A). Overall, we were mostly unanimous with our judgments, with some minor discrepancies that were discussed until we reached final decisions.

## 3 Results

In the initial search, after duplicates were removed, 336 articles were screened (See Fig. 1). Twelve duplicates were removed using Rayaan. A total of 29 full-text articles were sought for retrieval, of which 9 were included in the synthesis. In line with the PICO-informed inclusion criteria (See Table 1), 4 studies were excluded for having an irrelevant population, 9 were excluded for irrelevant intervention, 1 was excluded for not having a comparator, and 6 were excluded for irrelevant outcome measures. In the additional search, 56 articles were screened, 10 were sought for full-text retrieval, of which 2 were added to the synthesis. 1 study was excluded for having an irrelevant population, 3 were excluded for irrelevant intervention, and 4 were excluded for having no comparator. Thus, the total number of articles in the review and synthesis was 11.

### 3.1 Characteristics of included studies

The final sample consisted of eleven studies. The included studies were first categorized based on the sample, intervention characteristics, study design, and outcome measures (see Table 2).

Studies were conducted in Ireland (*N* = 1, Barrett and Stewart, 2021), the USA (*N* = 1, Fiery, 2016), Switzerland and Germany (*N* = 1, Hofer et al., 2018), Finland (*N* = 2, Lappalainen et al., 2013; Puolakanaho et al., 2020), China (*N* = 2, Lu et al., 2023; Zhang et al., 2024), Iran (*N* = 1, Otared et al., 2021), the UK (*N* = 1, Taylor et al., 2023), Canada (*N* = 1, Vahabi et al., 2022) and Japan (*N* = 1, Sasaki et al., 2023). Studies were mainly centered around high-income and upper-middle-income countries, particularly in Europe, North America, and East Asia. The studies were primarily conducted in Western nations (Ireland, Switzerland, Germany, the USA, Finland, the UK, and Canada) and East Asian countries (China and Japan), with some representation from the Middle East (Iran).

The studies differed in the participants' basic condition, sample size, measurement tools, and follow-up. The largest sample was Sasaki et al. (2023) (*N* = 841), and the smallest was Lappalainen et al. (2013) (*N* *=* 24)*.* The total sample size across studies was 2040. All studies included in the review reported the impact of an ACT-based intervention and measured outcomes at both pre- and post-intervention time points. Most studies (*N* = 9) also included follow-up assessments. Follow-ups were conducted at 1 month in two studies (Taylor et al., 2023; Zhang et al., 2024), 6 weeks in one study (Vahabi et al., 2022), 3 months in three studies (Hofer et al., 2018; Lu et al., 2023; Lappalainen et al., 2013), 6 months in three studies (Lappalainen et al., 2013; Puolakanaho et al., 2020; Sasaki et al., 2023), and 12 months in one study (Puolakanaho et al., 2020).

Inclusion criteria also varied substantially between studies. Five studies required participants to score above average (against normative data) on stress, burnout, depression, or anxiety measures (Hofer et al., 2018; Lu et al., 2023; Puolakanaho et al., 2020; Taylor et al., 2023; Sasaki et al., 2023). One study required participants to exhibit anxiety and depression symptoms based on diagnostic interviews (Otared et al., 2021). One study included participants with symptoms of stress, exhaustion, or sleeping problems (Lappalainen et al., 2013). One study required participants not to have a diagnosed mental health condition or be receiving psychological intervention (Fiery, 2016). Three studies did not specify any mental health-related inclusion criteria (Barrett and Stewart, 2021; Vahabi et al., 2022; Zhang et al., 2024).

### 3.2 Participant demographics

The ages of participants were relatively homogenous (*M* = 39.2, *SD* = 4.1), ranging between 33.4 and 46.9 years. Most studies (*N* = 10 out of 11 studies) reported participant gender (see Table 2 for a summary). Out of those who reported gender (*N* = 2009), all participants identified within the gender binary category: 83.3% identified as female and 16.7% identified as male. Notably, in their reporting, only Fiery (2016) explicitly listed non-binary/other options (e.g., Gender neutral/ Gender queer), but it is unsure whether this is due to most studies only asking to categorize gender within the male-female categorization, or whether this was not reported as no one identified as outside of the gender binary. Ethnicity was reported in far fewer studies (*N* = 2 out of 11 studies). Out of those who reported ethnicity (*N* = 521), overall, 88.3% were White, 4.6% were Asian, 2.7% were Black, 2.5% were mixed, and 1.9% were reported as “Other”. For a breakdown of the populations included for each study, see Table 2.

### 3.3 Narrative synthesis of results

Addressing aim 1, the following section synthesizes evidence on the effects of online ACT interventions in workplace settings on depression, anxiety, burnout, and stress, and assesses the methodological quality of studies.

#### 3.3.1 Burnout

Seven studies explored the effects of the respective interventions on burnout. Four studies reported significant between-group decreases in burnout in favour of the intervention group (Hofer et al., 2018; Lu et al., 2023; Puolakanaho et al., 2020; Zhang et al., 2024). Two found only within-group improvements with no significant difference compared to a CBT comparator (Barrett and Stewart, 2021) or waitlist control group (Lappalainen et al., 2013). One study reported no significant differences in burnout (Fiery, 2016).

In follow-up measures, Zhang et al. (2024) report between-group effects sustained at 1-month follow-up, Hofer et al. (2018) and Lu et al. (2023) found between-group effects sustained and strengthened at 3-month follow-up, Lappalainen et al. (2013) found within-group effects sustained and strengthened at 6-month follow-up, Puolakanaho et al. (2020) report that between-group effects were sustained at 6-month follow-up and strengthened at 12-month follow-up, Fiery (2016) observed no effects at 6-week follow-up relative to control.

#### 3.3.2 Depression

Eight studies explored the effects of the respective interventions on depression. Six studies found significant reductions in depression in the ACT group compared to control immediately at post-intervention (Hofer et al., 2018; Lu et al., 2023; Otared et al., 2021; Puolakanaho et al., 2020; Taylor et al., 2023; Zhang et al., 2024). Two studies reported non-significant effects (Lappalainen et al., 2013; Vahabi et al., 2022).

In follow-up measures, Zhang et al. (2024) report between-group effects sustained at 1-month follow-up, Taylor et al. (2023) found that effects were sustained but attenuated at 1-month follow-up, Hofer et al. (2018) and Lu et al. (2023) found between-group effects sustained and strengthened at 3-months follow-up, Puolakanaho et al. (2020) report that between-group effects were sustained at 6- and 12-months follow-up, Lappalainen et al. (2013) found within-group effects sustained at 6-months follow-up, and Vahabi et al. (2022) found no significant effects at 6-week follow-up.

#### 3.3.3 Anxiety

Six studies explored the effects of the respective interventions on anxiety. Five studies reported significant reductions in anxiety symptoms in the ACT intervention groups compared to controls immediately at post-intervention (Lu et al., 2023; Otared et al., 2021; Puolakanaho et al., 2020; Taylor et al., 2023; Zhang et al., 2024). One study found no significant group differences in anxiety scores (Vahabi et al., 2022).

In follow-up measures, Zhang et al. (2024) report between-group effects sustained at 1-month follow-up, Taylor et al. (2023) found that effects were sustained but attenuated at 1-month follow-up, Lu et al. (2023) found that between-group effects were sustained at 3-month follow-up, Puolakanaho et al. (2020) report that between-group effects were sustained at 6- and 12-month follow-up, and Vahabi et al. (2022) found no significant effects at 6-week follow-up.

#### 3.3.4 Stress

Seven studies explored the effects of the respective interventions on stress. Four studies reported significant reductions in stress in the ACT group compared to control immediately at post-intervention (Barrett and Stewart, 2021; Hofer et al., 2018; Puolakanaho et al., 2020; Zhang et al., 2024). Two studies found no significant between-group differences (Fiery, 2016; Vahabi et al., 2022). Sasaki et al. (2023) found no significant between-group effect in the intention-to-treat analysis, although a significant reduction was observed in the per-protocol analysis.

In follow-up measures, Zhang et al. (2024) found that between-group effects sustained at 1-month follow-up, Hofer et al. (2018) found between-group effects sustained and strengthened at 3-month follow-up, Puolakanaho et al. (2020) reported that between-group effects were sustained at 6- and 12-month follow-up, Vahabi et al. (2022) observed a within-group effect at 6-week follow-up, Fiery (2016) observed no effects at 6-week follow-up relative to control. Sasaki et al. (2023) reported no significant effects in intention-to-treat analyses at post-intervention or follow-up, although per-protocol analyses indicated a significant between-group effect at 3-month follow-up that was no longer significant at 6 months.

#### 3.3.5 Long-term effects

Several studies noted that meaningful change may emerge gradually rather than immediately post-intervention, consistent with ACT's emphasis on cumulative effects over time. Most studies incorporated relatively short follow-up measurements, which often demonstrated significant potential. For example, Lu et al. (2023) (3-month follow-up) and Puolakanaho et al. (2020) (12-month follow-up) observed some effects strengthened at follow-up compared to post-intervention. Similarly, Zhang et al. (2024) (1-month follow-up), Hofer et al. (2018) (3-month follow-up), and Taylor et al. (2023) (1-month follow-up) found that intervention effects persisted. Sasaki et al. (2023) (6-month follow-up) found that psychological distress significantly declined in participants who completed the intervention (per-protocol analysis), but not in the full sample (intention-to-treat analysis). Vahabi et al. (2022) found no significant outcomes, except for stress that was significant at the 6-week follow-up. Notably, Fiery (2016) did not find any significant outcomes at any time point, and Barrett and Stewart (2021) did not include follow-up measures. In short, follow-ups were generally short (typically less than a year), and one of the most frequent future research recommendations was longer follow-up periods, indicating that longer follow-ups could more comprehensively evaluate change.

### 3.4 Risk of bias assessment

**Fig. 2:**
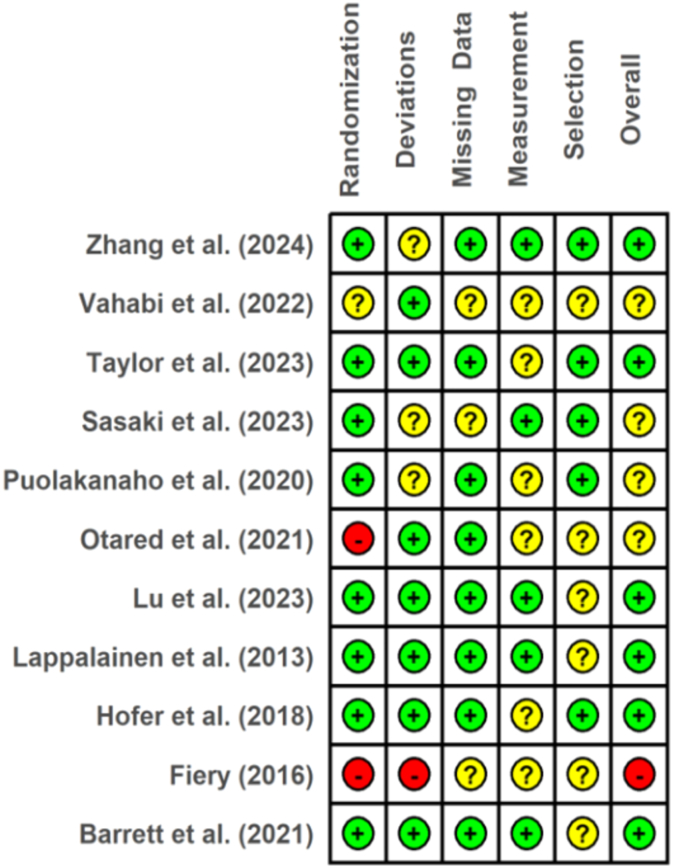
Cochrane risk of bias traffic plot. *Note.* Green = Low risk, yellow = Some concerns, red = High risk.

Taken together, no single risk-of-bias domain showed a consistent high-risk pattern across the evidence base. The main threats to certainty arise from (i) attrition and incomplete handling of missing data in a subset of trials and (ii) reliance on self-reported outcomes without participant blinding, which is inherent to this field. Conversely, trials with pre-registration, balanced randomization, appropriate longitudinal/ITT analyses, and higher retention offer greater confidence in their estimates (e.g., Hofer et al., 2018; Taylor et al., 2023; Zhang et al., 2024; Lu et al., 2023). Fig. 2 visually summarizes domain-level judgments, with original assessments by all three assessors provided in Appendix A.

#### 3.4.1 Randomization and allocation concealment

Randomization procedures were described in most studies and generally yielded balanced baseline characteristics (e.g., Taylor et al., 2023; Hofer et al., 2018; Lu et al., 2023; Puolakanaho et al., 2020). However, allocation concealment was sometimes vaguely reported, introducing uncertainty about selection bias in several trials (e.g., Lappalainen et al., 2013; Otared et al., 2021; Puolakanaho et al., 2020). In contrast, more robust procedures (e.g., block randomization via platform tools and clear descriptions of sequence generation) were evident in Taylor et al. (2023), and Zhang et al. (2024) reported blinded sequence generation and blinded data collectors, reducing risk in this domain.

#### 3.4.2 Deviations from intended interventions

Given the intervention type, participants were not blinded to assignment, which is typically unavoidable in behavioral trials (as opposed to drug trials). Despite this, there was no evidence of systematic protocol deviations. Automated or app-delivered formats likely limited provider influence (e.g., Taylor et al., 2023). Where active comparators were used, credibility was balanced (e.g., Barrett and Stewart, 2021), which may have mitigated expectancy differences between arms.

#### 3.4.3 Missing outcome data

Attrition varied considerably across studies. Several trials reported high retention and appropriate handling of missingness using ITT or longitudinal models (e.g., Lu et al., 2023; Hofer et al., 2018; Taylor et al., 2023). Others showed problematic or asymmetric dropout and limited sensitivity analyses (e.g., Vahabi et al., 2022). Fiery (2016) experienced extreme attrition (86.7% in the intervention group), making bias from missing data highly likely despite ITT, and was therefore judged high risk overall. Several studies explicitly examined dropout comparability (e.g., Lu et al., 2023; Zhang et al., 2024), which reduces concern that missingness depended on true outcomes.

#### 3.4.4 Measurement of outcomes

The relevant outcomes for this review (burnout, depression, anxiety, stress) were assessed using validated self-report questionnaires. This is standard practice in mental health trials, but it does carry a risk of expectancy or detection bias because participants were aware of their treatment allocation. A few studies took steps to reduce this risk, for example, by using blinded outcome assessors for follow-up data collection (Zhang et al., 2024; Sasaki et al., 2023). At the same time, the consistent use of validated instruments and standardized online administration across study arms likely minimized systematic differences in how outcomes were measured between groups.

#### 3.4.5 Selection of reported results

Pre-registration and analysis plans were unevenly reported across the included trials. Several studies demonstrated good transparency by registering protocols and aligning analyses with pre-specified plans (e.g., Lu et al., 2023 et al.; Hofer et al., 2018 et al.; Taylor et al., 2023 et al.; Zhang et al., 2024; Puolakanaho et al., 2020), while others did not explicitly report a registration or statistical analysis plan, leaving some uncertainty about selective outcome reporting (e.g., Lappalainen et al., 2013; Vahabi et al., 2022; Fiery, 2016). Thus, preregistration was present in part of the evidence base but not applied consistently across studies.

#### 3.4.6 Methodological quality and outcomes

Methodological rigor appeared to slightly co-vary with outcomes. Studies rated as having overall low risk of bias (e.g., Hofer et al., 2018; Lu et al., 2023; Taylor et al., 2023; Zhang et al., 2024) consistently reported significant between-group effects across multiple outcomes. In contrast, Fiery (2016), which showed very high attrition and was judged to have a high overall risk of bias, found null effects across outcomes. Several studies with “some concerns” in risk of bias also reported significant effects, indicating that while higher methodological rigor tends to co-occur with clearer positive findings, this relationship is not perfectly consistent.

## 4 Intervention delivery, engagement, and contexts

To address aim 2, the following section examines how these interventions have been delivered and studied.

#### 4.1.1 Barriers to engagement

Investigating the effectiveness of online interventions relies on maintaining participant engagement to enable meaningful comparisons. Engagement frequently emerged as a key concern limiting statistical power and analytic robustness. While online interventions provided flexibility (e.g., flexible time periods to go through intervention content), participants are simultaneously required to independently allocate time for participation, which presented challenges. A common reason for dropouts in the studies and across different occupational fields was a lack of time and too high workload (e.g., Barrett and Stewart, 2021; Fiery, 2016; Lu et al., 2023; Sasaki et al., 2023; Puolakanaho et al., 2020). Despite their flexibility, it appears that participants across occupational fields simply struggle to find sufficient time to engage with online interventions that are administered on top of their normal workload. For instance, Sasaki et al. (2023) reported that 48% of participants found the intervention too time-consuming.

Thus, participation may be perceived as an additional burden and could even lead to counterproductive experiences. Puolakanaho et al. (2020) found that participants who dropped out during recruitment reported higher burnout than those who continued, although most participants who started the intervention completed it. This suggests that when strain is already high, even the idea of an additional intervention, although targeted to improve well-being and reduce burnout, may feel too overwhelming.

#### 4.1.2 Engagement patterns

Across studies, attrition rates varied substantially, reflecting two types of intervention formats: self-guided and partially guided. Self-guided formats involved completely independent engagement with materials, such as pre-recorded materials (e.g. videos, audio) or other static materials (e.g. book, quizzes), without live interaction. Partially guided formats included self-guided materials alongside live interaction, such as facilitated therapist-led sessions with peer group discussions (See Table 4).

Self-guided interventions (Barrett and Stewart, 2021; Fiery, 2016; Hofer et al., 2018; Lu et al., 2023; Taylor et al., 2023; Sasaki et al., 2023) showed highly variable dropout rates among participants randomly assigned to the intervention condition. For example, Fiery (2016) and Sasaki et al. (2023) reported high attrition rates of 70.0% and 65.8%, respectively. In contrast, Hofer et al. (2018) and Lu et al. (2023) reported much lower rates of 13.1% and 5.5%, respectively. Barrett and Stewart (2021) had a moderate attrition rate of 40.9%, and Taylor et al. (2023) reported 11.0%.

Partially guided interventions (Lappalainen et al., 2013; Puolakanaho et al., 2020; Vahabi et al., 2022; Otared et al., 2021; Zhang et al., 2024) generally showed slightly lower (although still varying) dropout rates. For example, Lappalainen et al. (2013) and Puolakanaho et al. (2020) reported dropout rates of 9.1% and 17.0%, respectively. Vahabi et al. (2022) also had a low attrition rate of 5.8%, and Otared et al. (2021) did not report any attrition from the intervention group. However, Zhang et al. (2024) showed a higher rate of 35.2%, indicating that even partially guided formats may face adherence challenges.

Comparing these two, the overall weighted average of attrition for partially guided interventions was 18.9%, whilst the overall weighted average of attrition for self-guided interventions was 47.8%. Thus, partially guided interventions appeared to have slightly higher engagement overall. Engagement in self-guided interventions is varied and may be weaker without additional support. By contrast, partially guided formats achieved better retention, suggesting that even minimal human interaction (e.g., check-ins, peer support) may play a critical role in sustaining engagement.

With respect to the level of intervention guidance and outcomes, results were heterogeneous in both, indicating that the level of guidance did not guarantee better intervention outcomes. Among partially guided interventions, three of five trials (Puolakanaho et al., 2020; Otared et al., 2021; Zhang et al., 2024) demonstrated clear significant between-group effects, whereas two smaller-sample studies (*N* = 24–29) reported only non-significant or within-group findings (Lappalainen et al., 2013; Vahabi et al., 2022). Among self-guided interventions, three of six trials showed significant between-group effects (Hofer et al., 2018; Lu et al., 2023; Taylor et al., 2023), two showed mixed or conditional effects (Barrett and Stewart, 2021; Sasaki et al., 2023), and one showed null effects (Fiery, 2016).

### 4.2 Contextual influences

While the structure and delivery format of interventions can shape engagement, these effects were also embedded within broader occupational and cultural contexts that varied across studies. Occupational demands and cultural norms may shape both the engagement and perceived relevance of ACT interventions, making it difficult to generalize findings across sectors and regions without accounting for contextual factors.

#### 4.2.1 Recruitment strategies

The interventions were generally not reported to be embedded in broader health or well-being programs provided by the employer. In two studies, employers appeared to play a limited role in recruitment, as participants were recruited via (1) company-based recruitment, where human resources or occupational health departments distributed information about the study, and (2) individual self-enrolment through online advertisements (Puolakanaho et al., 2020; Sasaki et al., 2023). Other studies recruited participants via individual recruitment (e.g., online advertisements or community outreach), without reported employer awareness or involvement. In these cases, participation was situated entirely outside organizational systems, and the interventions were framed as independent programs.

#### 4.2.2 Occupational demands

The samples included diverse occupational contexts. Because of this heterogeneity, it is not possible to confidently disentangle in detail how each specific context influenced outcomes. Instead, the synthesis highlights broad contextual patterns that may help explain variation across studies. The working populations in half of the studies were listed as various sectors from the general working population (*N* = 6) (Fiery, 2016; Hofer et al., 2018; Lappalainen et al., 2013; Taylor et al., 2023; Puolakanaho et al., 2020; Sasaki et al., 2023), and just under half as frontline workers (*N* = 4), indicating healthcare staff and social workers (Barrett and Stewart, 2021; Lu et al., 2023; Otared et al., 2021; Zhang et al., 2024), and one study having migrant live-in caregivers (Vahabi et al., 2022). Participants across different occupational fields likely faced distinct types and intensities of job demands. For instance, Otared et al. (2021) and Lu et al. (2023) investigated health care workers during the COVID-19 pandemic, who may have had vastly different (although no less significant) occupational demands than, for instance, those in Lappalainen et al. (2013), who investigated a sample of employees from various occupational sectors in the early 2010s.

Across all occupational contexts, outcome patterns were heterogeneous. Among general working population samples, several studies reported significant between-group effects (Hofer et al., 2018; Puolakanaho et al., 2020; Taylor et al., 2023), while others showed only within-group improvements (Lappalainen et al., 2013) or benefits limited to per-protocol analyses (Sasaki et al., 2023). In frontline and healthcare samples, predominantly significant effects were observed across outcomes (Lu et al., 2023; Otared et al., 2021; Zhang et al., 2024; Barrett & Stewart, 2020), whereas studies in more niche occupational groups reported weaker or null effects (Fiery, 2016; Vahabi et al., 2022). Taken together, these findings indicate overall mixed outcomes across occupational contexts, with no consistent pattern that can be definitively attributed to population type alone, particularly given the small number of studies within each subgroup.

#### 4.2.3 ACT in wider cultural contexts

Many included studies were conducted in Western countries, where ACT was originally developed, and thus may reflect cultural assumptions of individualism, autonomy, and self-directed growth. A smaller number of studies in East Asian and Middle Eastern contexts (e.g., Lu et al., 2023; Zhang et al., 2024; Sasaki et al., 2023; Otared et al., 2021) indicate that while ACT processes remain broadly applicable, cultural orientation can shape how interventions are received. Sasaki et al. (2023) noted that their sample of Japanese working mothers (embedded in a collectivist cultural context) were less likely to prioritize autonomy-oriented values, underscoring the risk that protocols developed in Western settings may have limited cultural resonance when imported without adaptation.

Importantly, this issue reflects how ACT is applied rather than theoretical incompatibility. The core construct of psychological flexibility is sufficiently broad to encompass both individualist and collectivist orientations, as values within ACT can be defined in individual, relational, or collective terms. Challenges may arise when autonomy is emphasized more than relational or communal commitments. Thus, it appears that ACT's theoretical foundations may be culturally flexible but framing and delivery are critical for maximizing relevance.

## 5 Psychological flexibility and change

Addressing aim 3, we explore how psychological flexibility was conceptualized in the included interventions in relation to intervention-related change. ACT is grounded in the concept of psychological flexibility, making it important to examine how this process was addressed across the studies.

Despite contextual considerations, and consistent with the principles of ACT, psychological flexibility emerged as a central construct in most interventions. All but one study (Taylor et al., 2023) measured psychological flexibility in some way. Most studies quantified psychological flexibility using the Acceptance and Action Questionnaire (AAQ-II; Bond et al., 2011) (e.g. Barrett and Stewart, 2021; Hofer et al., 2018; Lappalainen et al., 2013; Lu et al., 2023; Otared et al., 2021; Puolakanaho et al., 2020; Vahabi et al., 2022) or the Work-related Acceptance and Action Questionnaire (Bond et al., 2013; Barrett and Stewart, 2021; Fiery, 2016) as the primary measure. Zhang et al. (2024) utilized the compACT (Călinici and Călinici, 2021), a slightly more recent process-based measure of psychological flexibility. Sasaki et al. (2023) measured psychological flexibility using an Euthymia Scale (adjusted for Japanese contexts; Sasaki et al., 2021), a transdiagnostic well-being measure consisting of psychological flexibility, a unifying outlook on life, and resistance to stress.

Additionally, two studies targeted specific subprocesses, mainly cognitive defusion or values alignment (Lu et al., 2023; Vahabi et al., 2022). Studies also frequently discussed the subprocesses of psychological flexibility as a key element of the intervention, with values-based work and cognitive defusion most frequently emerging as central processes. Overall, psychological flexibility was treated in broadly similar ways across studies, with only minor differences in emphasis.

### 5.1 Psychological flexibility as a mediator of change

Most studies positioned psychological flexibility as a primary explanatory factor for change, asserting that developing an individual's psychological flexibility is the mechanism that explains changes in the outcome measures. In this view, psychological flexibility was not only a contributing factor but also a central mechanism explaining why interventions worked (e.g., Zhang et al., 2024; Otared et al., 2021). For instance, Puolakanaho et al. (2020) explicitly explored psychological flexibility's role in mediating changes in burnout-related ill-being, with mediation analyses demonstrating that psychological flexibility consistently mediated group differences and outcome changes in work-related ill-being. Similarly, Lu et al. (2023) found that changes in psychological flexibility, and more specifically cognitive defusion and values-driven behavior, significantly mediated changes in burnout and depression. Although Barrett and Stewart (2021), Lappalainen et al. (2013), and Hofer et al. (2018) did not conduct mediation analyses, it is suggested that psychological flexibility might play a mediating role. These suggest that interventions aimed at improving psychological flexibility may be effective precisely because they target the mechanisms that mediate changes in occupational well-being.

### 5.2 Psychological flexibility as a moderator or outcome of change

One study positioned psychological flexibility as a moderator of intervention-related change, rather than a direct mechanism. Fiery (2016) examined whether psychological flexibility moderated the relationship between emotional job demands and emotional exhaustion. That is, instead of psychological flexibility being the mechanism that explains the change, psychological flexibility is viewed as a skill that can regulate how much job demands can affect emotional exhaustion. Although psychological flexibility was associated with lower emotional exhaustion, no significant moderation effect was found on the relationship between emotional job demands and emotional exhaustion, and the hypothesis of psychological flexibility playing a moderating role was not supported.

Other studies positioned psychological flexibility as an outcome of intervention change, rather than as a direct mechanism. For instance, Sasaki et al. (2023) assessed psychological flexibility as an intervention target rather than a mediator, and psychological flexibility significantly increased among participants. Vahabi et al. (2022) included flexibility within their outcome battery, although results were largely non-significant. This is not to suggest that these studies rejected psychological flexibility as driving change, but rather that this was not their central focus in investigating intervention changes.

## 6 Discussion

This systematic narrative review synthesized findings from 11 RCTs that investigated online ACT-based interventions in occupational contexts. First, we investigated the effectiveness of the interventions on burnout, depression, anxiety, and stress. Second, we explored how these interventions have been delivered and studied, and then how psychological flexibility was conceptualized in relation to intervention-related change. Finally, in this discussion and addressing aim 4, we identify strengths, limitations, and research gaps in the current literature.

Based on the current evidence, online ACT-based interventions show tentative potential in reducing employee burnout, depression, anxiety, and stress, but the findings are inconsistent and derived from a relatively small number of heterogeneous trials. Four out of seven studies reported significant between-group reductions in burnout, six (6/8) in depression, five (5/6) in anxiety, and four (4/7) in stress.

### 6.1 Strengths, limitations, and research gaps

The literature on the topic remains relatively scarce, as reflected in the small number of studies meeting the inclusion criteria. This aligns with previous reviews investigating (non-online) ACT-based interventions in occupational contexts, which highlight that while such interventions are promising, ACT is still more frequently utilized in clinical settings and the evidence base is still growing within occupational contexts (Towey-Swift et al., 2023; Vega-Campos et al., 2025).

The results somewhat mirror wider findings; a meta-analysis of in-person workplace ACT studies found that ACT significantly outperformed control conditions in reducing psychological distress and stress and overall well-being, although effects were generally small to moderate and accompanied by substantial heterogeneity (Unruh et al., 2022). Similarly, a review on professional burnout reported that in-person ACT-based interventions led to measurable reductions in burnout across most studies, although inconsistent protocols precluded definitive conclusions (Towey-Swift et al., 2023). A meta-analysis evaluating online ACT in clinical contexts similarly reported significant but small between-group effects of ACT on anxiety and depression, although results were mixed when compared to active control groups (Klimczak et al., 2023).

One reason for the inconsistent effects can be that the interventions were delivered primarily online and either with a fully self-guided or partially guided structure, with considerable variation in engagement. Partially guided interventions showed lower attrition (weighted average = 18.9%) than self-guided interventions (weighted average = 47.8%), which is consistent with previous research indicating that guided digital mental health interventions tend to have higher engagement than self-guided interventions (Borghouts et al., 2021). Low engagement in self-guided online well-being interventions is widely noted as a substantial issue in their effective delivery (Gulliver et al., 2021), and even with a guided element, online mental health and well-being interventions may easily overwhelm users, contributing to dropout (Cross and Alvarez-Jimenez, 2024).

Although partially guided interventions showed better adherence than self-guided, this did not consistently translate into superior outcomes. This suggests that guidance may primarily support engagement rather than guarantee stronger clinical effects. Importantly, the partially guided studies reporting null or mixed effects (Lappalainen et al., 2013; Vahabi et al., 2022) were also those with the smallest sample sizes (*N* = 24–29), indicating that insufficient statistical power is a plausible explanation for these null findings.

Interestingly, a few studies in this review observed that the benefits of ACT-based interventions often continued to develop after the intervention. For example, Lu et al. (2023) reported stronger effects at a three-month follow-up, and Puolakanaho et al. (2020) noted greater improvements even at 12 months. This pattern aligns with ACT's theoretical framework (e.g. Hayes et al., 2006), which emphasizes the gradual development of psychological flexibility and the cumulative impact of behavioral changes. This is consistent with evidence from broader clinical contexts indicating that the benefits of online ACT can persist or strengthen over time (e.g. Graham et al., 2025; Trindade et al., 2021).

Although most studies in the review incorporated follow-up measures, most of them were relatively short (less than six or twelve months). Short-term assessments may therefore underestimate the full impact of ACT, and extended follow-ups are important for evaluating sustained outcomes. Thus, future research should also include longer-term follow-ups to capture change more comprehensively.

The methodological heterogeneity of the reviewed studies, including considerable variability in the inclusion criteria of studies, limits the extent of conclusions. Some studies included only individuals experiencing elevated levels of psychological distress (e.g., Hofer et al., 2018; Lu et al., 2023), while others had minimal or no mental health-related screening (e.g., Barrett and Stewart, 2021; Zhang et al., 2024). This inconsistency likely introduced baseline differences in participants' symptoms, motivation for change, and responsiveness to the interventions, complicating direct comparisons. Although outcome measures were pre-specified in this review, studies varied in how they assessed burnout, depression, anxiety, and stress, often using different tools with distinct subscales, thresholds, and formats, which complicates the strength of conclusions.

These inconsistencies are still notable and not unique to this review. The field continues to lack coherence regarding how ACT is operationalized across studies, often without a clear specification of the intervention protocol and limited detail on delivery components and mechanisms of change (Towey-Swift et al., 2023; Vega-Campos et al., 2025). This points to a broader challenge in attempting to evaluate ACT primarily through RCT designs; while RCTs are essential for establishing causal efficacy (Hariton and Locascio, 2018), their focus on aggregated group-level outcomes may restrict insight into how interventions work or for whom they are most effective (Kazdin, 2007; Hayes et al., 2019). Recent work underscores that ACT does not operate as a monolithic package but through the subprocesses of psychological flexibility, which may be activated differentially across individuals (Rad et al., 2025). From this perspective, the mixed findings may not only reflect variability in intervention design but also unmeasured individual differences in which processes were engaged.

Consequently, there have been calls for more precise, multidimensional approaches to assessing psychological flexibility and its subprocesses (Doorley et al., 2020; Cherry et al., 2021). Process-oriented measures such as Psy-Flex (Gloster et al., 2021) and the Process-Based Assessment Tool (PBAT; Ciarrochi et al., 2022) may provide greater conceptual clarity and allow closer examination of how different subprocesses operate over time. Incorporating such measures would help clarify the mechanisms of change, capture individual variability, and advance the field toward more nuanced, process-based evaluations of online ACT interventions in workplace settings.

Further limitations concern the lack of diversity in participant samples. Participants' demographics were largely homogenous in age (*M* *=* 39.2, *SD* = 4.1), but the sample was heavily skewed toward female-identifying participants (83.3%), ethnicity was rarely reported (2 out of 11 studies), and where ethnicity was reported, the sample was majority white (88.3%), limiting the broader applicability of findings. Although interventions were conducted in several countries, most were situated in high-income Western contexts, with limited representation from East Asia and the Middle East. Yet the available evidence suggests that cultural context may shape how ACT is received. For instance, Sasaki et al. (2023) found that in a Japanese sample, where collectivist values and relational roles were more salient, participants engaged more strongly with relational aspects of the intervention than with purely individual change. This highlights how cultural factors can influence not only the overall experience of ACT, but also which components resonate most.

ACT research and adaptations are growing rapidly worldwide, including in Latin America (Cañón et al., 2023) and Sub-Saharan Africa (Musanje et al., 2024), but remain disproportionately concentrated in high-income Western countries (Lim et al., 2024). This continued emphasis on Global North settings may restrict the broader cultural applicability of findings and highlights the need for more research in underrepresented regions, such as the African continent, Southeast Asia, Latin America, and areas of the Middle East. Again, this is not a novel limitation: Schimmelpfennig et al. (2024) emphasize that moving beyond WEIRD (Western, Educated, Industrialized, Rich, and Democratic) samples and adopting more context-sensitive, cross-cultural approaches is an essential future direction for understanding how interventions function across varying socio-ecological conditions. Individualist versus collectivist cultural norms, levels of inequality, collective experiences of threat, and historical context may all shape how ACT-based interventions are received and interpreted. Pragmatic adaptations, such as using culturally resonant metaphors, foregrounding relational values where relevant, and employing measures validated for cross-cultural equivalence, have been recommended to improve both engagement and effectiveness (Leung et al., 2024; Gloster et al., 2021). Future research should therefore move beyond testing ACT in new regions to systematically adapting its processes and delivery to cultural contexts.

### 6.2 Limitations of the synthesis

The synthesis is limited by a few key factors (Campbell et al., 2020). First, we conducted two searches, with the second taking place after reviewing the studies from the first search. While the first search was screened independently by three authors, the second search was carried out by one author, with final inclusion decisions discussed collaboratively between all authors. This introduces some risk of selection bias, as independent screening was not maintained throughout. Moreover, the second search involved a post hoc revision of the inclusion criteria to incorporate stress, which may affect consistency and replicability. Despite this, this decision was based on the first search, which produced a limited number of studies and where stress frequently emerged as a relevant concept, and thus the second search aimed to capture stress as well as a broader and more relevant evidence base, and we aimed for maximally transparent reporting regarding the second search. Therefore, although it is not an uncomplicated addition, the additional search likely still strengthened the review by including studies that would have otherwise been overlooked.

Second, due to the heterogeneity in study design, intervention format, and outcome measures, studies were grouped narratively by delivery format, occupational context, and cultural setting. While this structure supported cross-study comparison, it also risks oversimplifying differences within groups and underemphasizing alternative explanations for variation in outcomes. As such, the conclusions presented here reflect broad patterns rather than precise estimates and should be interpreted with caution in relation to the original review question.

### 6.3 Recommendations

Across the included studies, the interventions were not integrated into broader occupational structures or well-being initiatives, which may have limited their impact. The most common reasons for dropout appeared to be time constraints and heavy workloads, highlighting that even with accessible online formats, online interventions still face critical barriers. Thus, the interventions might lose meaning before they begin, as employees simply lack the time to engage, even if the content is theoretically well-matched to their needs. These challenges are particularly relevant given the growing expectation that employers take an active role in supporting workplace mental health (Stuber et al., 2021; Hammer et al., 2024). Overall, engagement barriers were primarily practical (time/workload) rather than motivational, underscoring the need for intervention designs that embed sessions into working hours, provide organizational support, or leverage brief “micro-interventions” to reduce perceived burden.

While interest in scalable interventions is rising (Fleming, 2024), the practical barriers identified in this review (time and organizational support) highlight the need for more integrated, system-level approaches to intervention delivery. This aligns with recent evidence showing that individual-level interventions alone often yield limited benefits unless combined with organizational changes that provide the time, resources, and legitimacy for participation (Fleming, 2024). It is increasingly relevant that employers do not simply “dump” such interventions on employees but actively support and create opportunities to engage properly.

Furthermore, this review suggests that guidance and/or peer support generally supports engagement in online interventions, with human contact adding a clear positive aspect to the interventions. Peer support was highlighted as a valuable opportunity for experiencing connection, social networking, observing inner processes, and recognizing personal reaction patterns (Lappalainen et al., 2013; Puolakanaho et al., 2020; Vahabi et al., 2022). These factors may give some context as to why attrition appeared to be slightly lower in partially guided interventions. Similarly, Borghouts et al. (2021) report that participants specifically valued the connection with others via guided online interventions as opposed to self-guided ones. More broadly, live coaching or peer support within non-ACT-based online programs has also been associated with participants' sense of connection, engagement, and perceived support in occupational contexts (Boß et al., 2025; Althammer et al., 2023). Thus, partially guided formats may combine relational and motivational benefits of live interaction with the accessibility of online delivery (Borghouts et al., 2021).

Nevertheless, self-guided interventions still demonstrate some promise if contextualized for the participant. For engagement, especially in self-guided contexts for high-stress occupational fields, tailoring the content to the specific occupational context may support engagement and positive outcomes. For instance, Fiery (2016) notes that their fully self-guided intervention was not contextualized for the animal shelter workers, which may have contributed to why participants struggled to engage and find the content motivating. Conversely, Barrett and Stewart (2021) specifically tailored intervention content to healthcare contexts, and engagement was more consistent and received well, although the intervention was fully self-guided. Previous research indicates that when participants feel more connected to the content, engagement is often high in self-guided interventions (van der Lubbe et al., 2023). Moreover, self-guided interventions can offer anonymity and privacy that may be harder to achieve in guided interventions to reduce fear of stigmatization (Gulliver et al., 2021).

## 7 Conclusion

This review indicates that online ACT-based interventions hold tentative promise for reducing burnout and depression, with more variable findings for anxiety and stress. Partially guided formats appeared to support engagement more effectively than fully self-guided approaches, although patterns were not consistent. The small number of heterogeneous trials limits the strength and generalizability of conclusions. We lack certain evidence, particularly regarding replication across independent studies, systematic evaluation in diverse cultural contexts, and long-term follow-up of outcomes. Future research should address these gaps through more standardized methodologies and reporting, the use of process-based measures of psychological flexibility, attention to individual differences by examining change in processes or sub-processes at the individual level, and culturally responsive adaptations to better identify what works, for whom, and under which conditions.

## Declaration of Generative AI and AI-assisted technologies in the writing process

During the preparation of this work, the authors used the University of Helsinki's CurreChat generative AI tool and ChatGPT (GPT-5.2) for clarity checks and to improve the readability of the paper by shortening a few paragraphs, and Grammarly for AI-assisted spell checks. After using these tools, the authors reviewed and edited the content as needed and take full responsibility for the content of the publication.

## Funding

This work was supported by Business Finland through the Research to Business (R2B) funding instrument (6444/31/2022) and by the 10.13039/501100003125Finnish Cultural Foundation (00250570). Open access funded by Helsinki University Library.

## Declaration of competing interest

The authors declare no conflicts of interest.

## Figures and Tables

**Figure:**
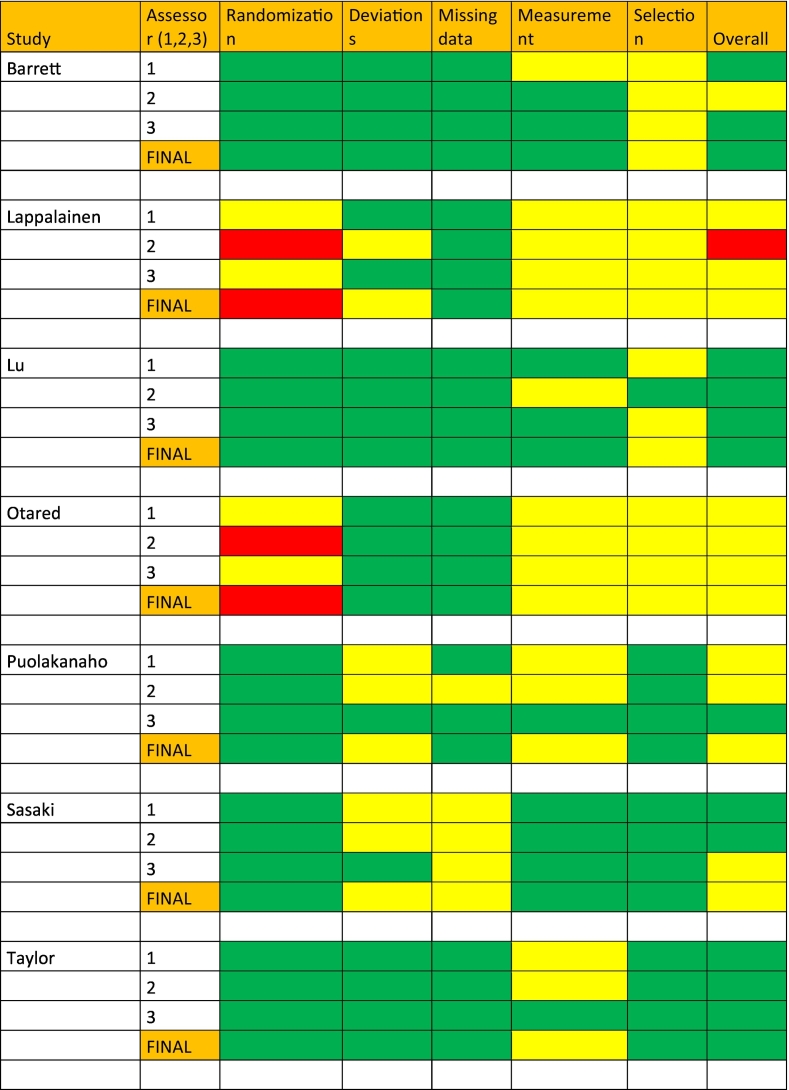

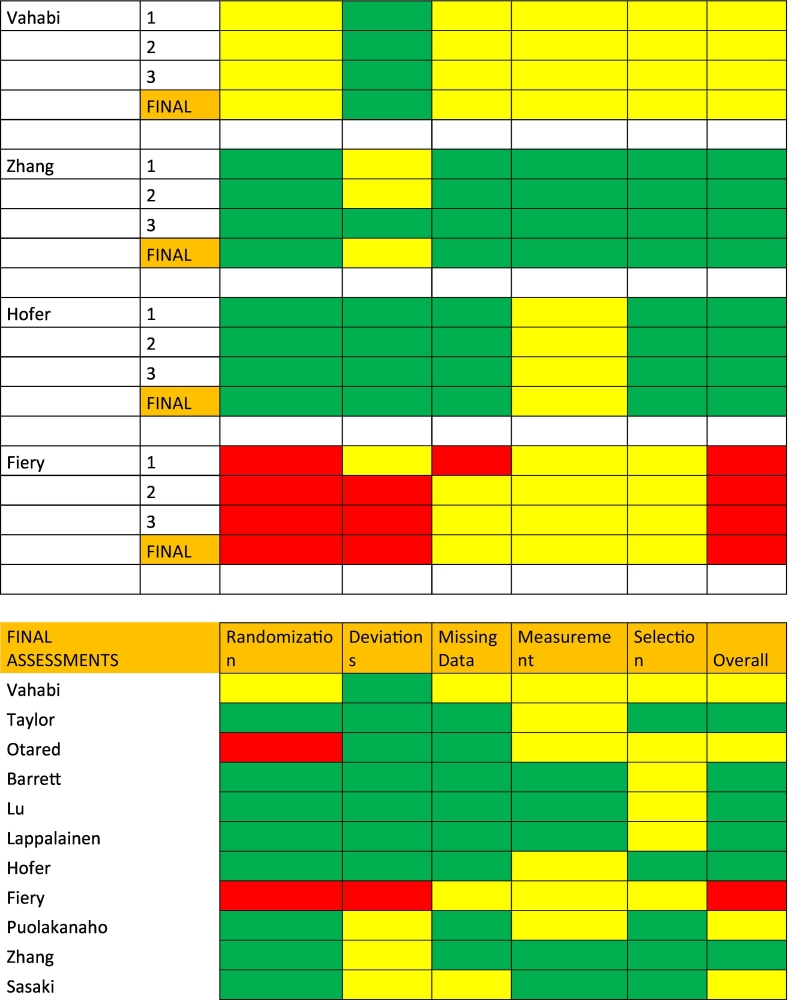


## Appendix B Full search strings for each search

In the initial search, the search terms were: (Online OR web OR internet OR online) AND (ACT OR Acceptance Commitment therapy OR Acceptance and Commitment Therapy) AND intervention AND psychological flexibility AND (burnout OR depression OR anxiety) AND (working life OR occupational OR work OR job OR professional OR profession OR worker OR employee) AND (RCT OR randomized controlled trial).

In the additional search, the search terms were: (Online OR web OR internet OR online) AND (ACT OR Acceptance Commitment therapy OR Acceptance and Commitment Therapy) AND intervention AND psychological flexibility AND (burnout OR depression OR anxiety OR stress) AND (working life OR occupational OR work OR job OR professional OR profession OR worker OR employee) AND (RCT OR randomized controlled trial).
